# Minimum Two-Year Outcomes of the Zimmer G7 Modular Dual Mobility Cup in Primary Total Hip Arthroplasty: Survivorship, Complications, Clinical and Radiographic Results

**DOI:** 10.3390/jcm14197071

**Published:** 2025-10-07

**Authors:** Marco Minelli, Vincenzo Longobardi, Vincenzo Paolo Di Francia, Alessio D’Addona, Marco Rosolani, Federico Della Rocca

**Affiliations:** 1 Department of Biomedical Sciences, Humanitas University, Via Rita Levi Montalcini 4, Pieve Emanuele, 20090 Milan, Italy; 2 IRCCS Humanitas Research Hospital, Via Manzoni 56, Rozzano, 20089 Milan, Italy

**Keywords:** arthroplasty, replacement, hip, dual-mobility, modular acetabular cup, primary total hip arthroplasty

## Abstract

**Background/Objectives:** Modular dual mobility (MDM) cups are constituted by a cobalt-chromium liner inserted into a standard acetabular shell, allowing for intraoperative indication and supplementary screw fixation of the acetabular component. MDM could face mechanical and biological issues, with the associated risk of elevated blood metal ions levels and adverse local tissue reactions. **Methods:** This is a monocentric retrospective study on a consecutive series of 105 patients who underwent primary unilateral THA with the G7 Dual Mobility Acetabular System cup (Zimmer Biomet, Warsaw, IN, USA) from March 2019 to April 2023, and who were evaluated clinically and radiographically at a minimum two-year follow-up. All complications and revisions were recorded. Survivorship analysis with any revision surgery as endpoint was performed using Kaplan–Meier survival curves. **Results:** There were eighty-nine patients (follow-up rate 84.8%) who underwent clinical and radiographic follow-up. The mean follow-up was 2.5 ± 0.8 years. Revision-free survival was 98.0%. Three complications (2.8%) were recorded: one case of posterior dislocation, one periprosthetic joint infection and one post-traumatic periprosthetic femur fracture. Dislocation rate and infection rate were less than 1.0%. None of the patients were revised for adverse local tissue reactions. No cup loosening was observed. No cases of intraprosthetic dislocation, liner malseating or femoral notching were observed. Retroacetabular stress shielding was present in 43.0% of patients. Clinical scores significantly improved at the last follow-up compared with preoperative status (*p* < 0.0001): the final mean mHHS was 87.5 ± 5.3 and the final mean VAS was 0.5 ± 0.9. **Conclusions:** The Zimmer G7 modular dual mobility cup appears to be a safe and effective option and does not present specific implant-related mechanical and biological issues in primary total hip arthroplasty at a minimum two-year follow-up.

## 1. Introduction

Total hip arthroplasty (THA) is one of the most successful orthopedic procedures, providing excellent pain relief and functional outcomes for patients with end-stage hip disease [1]. Demand for it is steadily increasing worldwide, with projections indicating a substantial rise in the coming decades due to aging populations and expanding indications [2]. Still, dislocation is one of the main complications of total hip arthroplasty [3]. In this context, dual mobility (DM) acetabular cups, first introduced by Bousquet in the 1970s, feature a distinctive dual articulation: one between the femoral head and the mobile polyethylene liner and one between the mobile polyethylene liner and the acetabular shell [4]. This configuration improves the head–neck ratio and increases both jump distance and impingement-free range of motion, thereby lowering the risk of dislocation [5]. Nonetheless, monoblock dual mobility cups were associated with specific complications, including intraprosthetic dislocation (IPD) and aseptic loosening [5,6]. In 2009, modular dual mobility (MDM) components were introduced in the US market [5]. Modular dual mobility acetabular cups are constituted by a liner which is inserted into a standard titanium acetabular shell, allowing for intraoperative indication and supplementary screw fixation of the acetabular component [4]. Modular dual mobility liners are generally made of cobalt-chrome (CoCr) [7]. Thus, modular dual mobility constructs could face mechanical and biological issues [8,9,10,11,12]. In particular, mechanical issues, such as femoral notching due to neck-rim impingement, liner malseating, iliopsoas tendinitis, and IPD were observed [8,9]. In addition, biological issues have been raised, particularly fretting corrosion at the liner–cup interface, which may generate cobalt–chrome debris and result in elevated metal ion levels with a risk of adverse local tissue reactions (ALTR) [10,11,12]. Thus, the MDM construct longevity and its systematic application in primary total hip arthroplasty is still debated [13]. The primary endpoint of this study was to report survivorship, complication and revision rates in a consecutive series of patients who received an MDM bearing for primary THA at a minimum two-year follow-up. The secondary endpoint was to evaluate the MDM acetabular system clinical and radiographic outcomes.

## 2. Materials and Methods

### 2.1. Study Design and Inclusion Criteria

**Figure 1:**
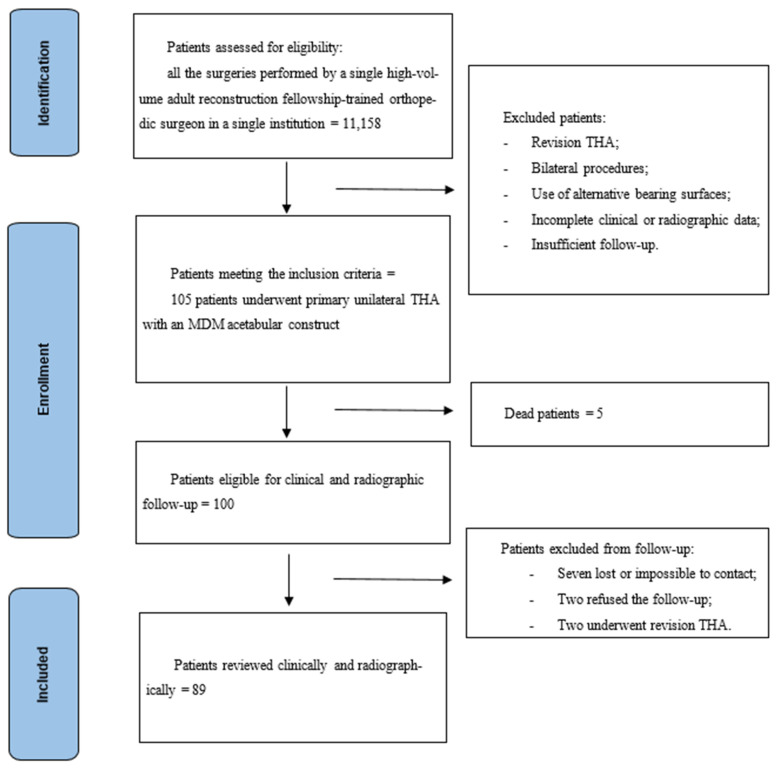
Study design and inclusion criteria.

This is a monocentric retrospective study on a consecutive series of patients who underwent total hip arthroplasty with modular dual mobility bearing. All the surgeries were performed by a single high-volume adult reconstruction fellowship-trained orthopedic surgeon. Inclusion criteria were primary THA, unilateral procedures, use of an MDM acetabular cup and a minimum two-year follow-up. Exclusion criteria were revision THA, bilateral procedures, use of alternative bearing surfaces, incomplete clinical or radiographic data, insufficient follow-up and patients unwilling or unable to provide informed consent or to participate in a follow-up. A total of 105 patients (32 men, 73 women) met the inclusion criteria and underwent primary unilateral THA with an MDM acetabular construct from March 2019 to April 2023. The mean age at surgery was 73.8 years (range 35.0–97.0 years). The surgical indications were primary osteoarthritis in 83 cases (79.0%), avascular necrosis in 11 patients (10.5%), post-traumatic osteoarthritis in 5 patients (4.8%), acute femur neck fracture in 2 cases (1.9%), high-dislocation Crowe IV adult hip dysplasia in 2 patients (1.9%), Girdlestone sequelae in one case (1.0%) and neck fracture sequelae in one patient (1.0%). After a minimum follow-up of two years, five patients died for causes unrelated to hip replacement, seven were lost or impossible to contact, two cases refused the control, and two patients were revised. In the case of death, the date and the eventual data of any surgical procedures related to the implant carried out in other hospitals were asked and recorded. At the end, 89 patients were reviewed clinically and radiographically at a mean follow-up of 2.5 years (range 2.0–6.0) (Figure 1). The reviewed patients were sixty-two women (69.7%) and 27 men (30.3%). Considering the reviewed patients, mean age at surgery was 73.6 years. The study was approved by the independent Institutional Ethics Committee of the IRCCS Humanitas Research Hospital (protocol number 618/17).

### 2.2. Surgical Procedure

**Figure 2:**
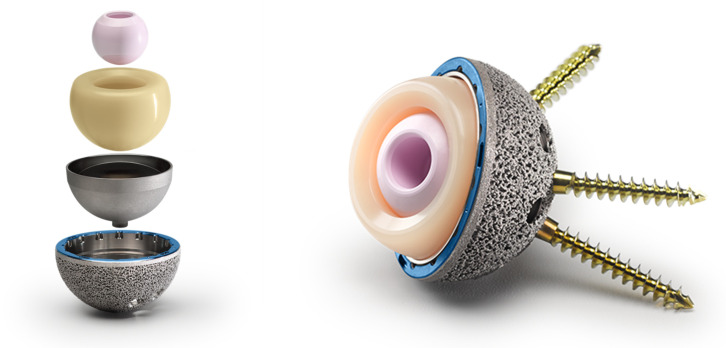
G7 Dual Mobility Acetabular System cup (Zimmer Biomet, Warsaw, IN, USA). Image reproduced with permission from Zimmer Biomet (Warsaw, IN, USA). All rights reserved.

Preoperative templating was carried out with the OsiriX DICOM Viewer (Pixmeo, Geneva, Switzerland): the femoral offset and lesser trochanter to head center distance were measured on the contralateral hip in order to intraoperatively achieve hip biomechanics reconstruction [14]. All the patients underwent the procedure under spinal anesthesia. Surgical exposure was performed through a postero-lateral approach for all the patients. A cementless G7 Dual Mobility Acetabular System cup (Zimmer Biomet, Warsaw, IN, USA) was implanted in all the cases (Figure 2). The acetabular bone was progressively reamed, starting from 6 to 8 mm smaller than the planned acetabular implant size up to 1 mm under the final implant size. The final shell anteversion was decided according to the “femur first technique”: the cup was positioned in an anteversion compliant to the definitive stem antetorsion [14]. Whenever intraoperative assessment raised concerns about press-fit stability or revealed suboptimal bone quality, supplementary screws were placed. Supplementary screws were implanted in 2 patients (1.9%). The distribution of implanted acetabular cup sizes ranged from 46 to 62 mm: 46 mm in 8 cases (7.6%), 48 mm in 27 patients (25.7%), 50 mm in 31 cases (29.5%), 52 mm in 16 patients (15.2%), 54 mm in 10 cases (9.5%), 56 mm in 6 patients (5.7%), 58 mm in 1 case (1.0%), 60 mm in 4 patients (3.8%), and 62 mm in 2 cases (1.9%). The acetabular cup coating was Porous Plasma Spray (PPS) in 67 cases (63.8%) and OsseoTi Porous Structure in 38 patients (36.2%). A highly porous titanium coating (i.e., OsseoTi Porous Structure) was used to facilitate bone ingrowth whenever acetabular bone quality was suboptimal. The appropriate CoCr dual mobility liner was chosen according to the size of the definitive acetabular shell. All the tapers of the shell were dried, any debris was removed, and it was checked for the apical plug to not be present at the dome of the shell. Then, the metal liner inserter ring was pressed onto the face of the definitive liner to align the taper interface for the proper seating of the CoCr liner into the shell and to avoid liner malseating. After having ensured an even contact of the liner inserter ring with the face of the shell, the definitive CoCr liner was impacted with the appropriate ball impactor. During impaction, the hard bearing inserter ring disengaged from the definitive liner and was removed. The CoCr dual mobility liner insert inner diameter sizes ranged from 36 to 48 mm. The distribution of liner diameter sizes was: 36 mm in 8 cases (7.6%), 38 mm in 27 cases (25.7%), 40 mm in 31 cases (29.5%), 42 mm in 16 cases (15.2%), 44 mm in 16 cases (15.2%), 46 mm in 5 cases (4.8%) and 50 mm in 2 cases (1.9%). Either a 22 mm or a 28 mm head size was used, depending on the acetabular shell size. Head size was 22 mm in 8 cases (7.6%) and 28 mm in 97 cases (92.3%). Overall, 8 femoral heads (7.6%) were made of CoCr and 97 (92.3%) were made of ceramic. A vitamin E-stabilized highly crosslinked polyethylene (Vivacit-E) mobile liner was used in all of the cases. Acetabular and head components are shown in Table 1. Femoral stems were cemented in 31 cases (29.5%) and cementless in 74 patients (70.5%); a standard cementless stem was used in 69 cases (65.7%) and a conical tapered stem was used in 5 cases (4.8%). The standard cementless femoral stems were 45 CLS Spotorno Hip Stem 135° neck stems and 24 CLS Spotorno Hip Stem 125° neck stems (Zimmer Biomet, Warsaw, IN, USA). Conical tapered cementless stems were 3 Zimmer Wagner Cone 135° neck stems and 2 Zimmer Wagner Cone 125° neck stems. Cemented stems were 31 Zimmer MS30 standard neck stems. Femoral stem components are shown in Table 2 and Table 3.

### 2.3. Clinical and Radiological Evaluation

Clinical evaluation was performed preoperatively and postoperatively and included the modified Harris Hip Score (mHHS) and the Visual Analog Scale (VAS) for pain [15,16]. All complications and reoperations were recorded.

At the last follow-up, all patients underwent standing radiographic assessment with the EOS 2D/3D imaging system (Biospace Med, Paris, France). Three-dimensional reconstructions of the skeletal system and prosthetic components were generated using dedicated software (sterEOS 3D, version 1.5.3.7947, Biospace Med, Paris, France) to measure acetabular inclination and combined anteversion. All the images were evaluated by two orthopedic specialists (A.D. and M.R.) trained in hip surgery. Whenever differences in assessment were noted, the observers reached a consensus. Inter-observer reliability was assessed using the intraclass correlation coefficient (ICC) and was 0.91, which is generally considered excellent [17]. Acetabular radiolucent lines (RLLs) were classified according to the DeLee and Charnley classification [18]. Acetabular cup loosening was considered to be present if the acetabular component had progressively migrated, as demonstrated by shift, tilt or subsidence, or if complete radiolucency ≥ 2 mm was present in each zone [19]. A periacetabular decrease in bone density compatible with retroacetabular stress shielding was observed [20]. Radiographically evident femoral notching and liner malseating were reported whenever present. Heterotopic calcifications were reported whenever present.

### 2.4. Data Analyses

Survivorship was analyzed with Kaplan–Meier curves using any revision as the endpoint. For patients lost to follow-up or deceased without a documented date of death, the last available follow-up was used. Continuous variables were summarized as mean ± standard deviation or as ranges (minimum–maximum), while categorical variables were expressed as counts and percentages. Complication and revision rates were reported as crude event proportions. To assess potential informative censoring, baseline characteristics were compared between patients reviewed at ≥2 years and those lost to follow-up or deceased. Continuous variables were compared using t-tests or Mann–Whitney tests, and categorical variables were compared using Chi-squared or Fisher’s exact tests. An exploratory subgroup analysis was conducted to compare the clinical and radiographic outcomes between different acetabular cup coatings. Normality of the data distribution was assessed using the D’Agostino–Pearson test. Depending on the distribution, either a paired t-test or the Mann–Whitney test was applied to evaluate statistical significance. Qualitative variables were compared using Chi-squared or Fisher’s exact tests. Statistical analysis was performed with EasyMedStat software (version 3.40; EasyMedStat, Paris, France; www.easymedstat.com; accessed on 27 March 2025).

## 3. Results

### 3.1. Survival Analysis, Complication and Revision Rates

**Figure 3:**
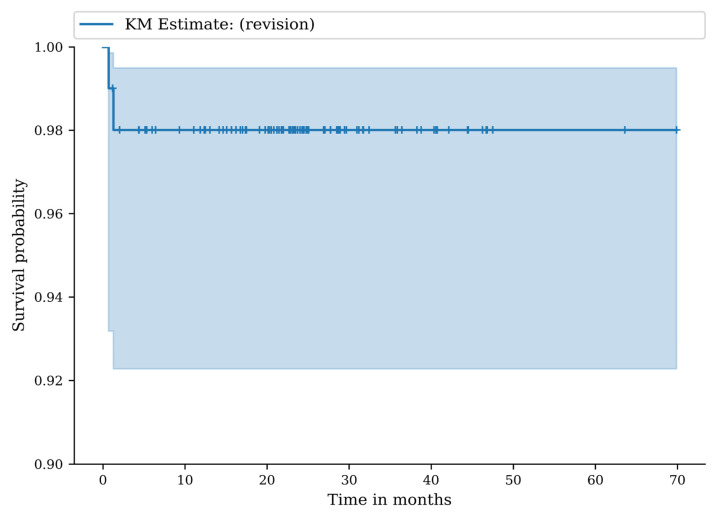
Kaplan–Meier (KM) survival curve with any revision of the implant as endpoint.

There were eighty-nine patients who were reviewed clinically and radiographically at a mean follow-up of 30 months (range 24–72). The follow-up rate was 84.8%. At 24 months, the revision-free survival was 98.0% (95% CI: 92.3–99.5), and at 36 months, the revision-free survival was 98.0% (95% CI: 92.3–99.5) (Figure 3).

Three complications were recorded (complication rate 2.9%). Specifically, one atraumatic posterior dislocation which underwent revision with short to medium head exchange, one post-traumatic periprosthetic diaphyseal femur fracture which stabilized after conservative treatment, and one *Pseudomonas aeruginosa* infection which was treated with a two-stage revision. Dislocation rate was 0.9%. Revision rate was 1.9%. In both cases, at least one component of the acetabular implant was removed, so the overall implant removal rate was 1.9%. None of the patients were revised for adverse local tissue reactions. No cases of intraprosthetic dislocation were reported. No periprosthetic cup fractures were observed.

### 3.2. Clinical Outcomes

All the clinical scores significantly improved at the last follow-up compared to pre-operative status, independently of the surgical indication. Preoperative mean mHHS was 37.6 ± 12.5 (range 7.0–77.0), postoperative mean mHHS was 87.5 ± 5.3 (range 69.0–91.0), *p* < 0.0001. Preoperative mean VAS was 8.4 ± 1.4 (range 6.0–10.0), postoperative mean VAS was 0.5 ± 0.9 (range 0–4.0), *p* < 0.0001. Among the 89 clinically reviewed patients, 5 cases reported to suffer from painful trochanteric syndrome (5.6%) and 1 patient (1.1%) was diagnosed with iliopsoas tendinitis. Subgroup analysis between PPS and OsseoTi cup coatings showed no significant differences in clinical outcomes.

### 3.3. Radiographic Outcomes

**Figure 4:**
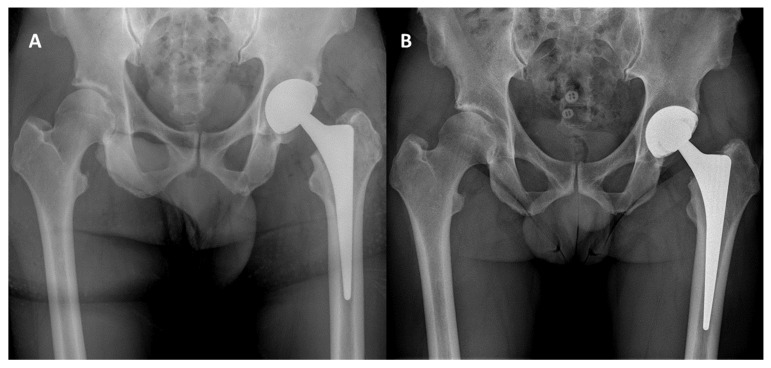
(**A**): Immediate postoperative radiographic control; (**B**): 2.5-year radiographic follow-up showing a decrease in periacetabular cancellous bone density with retention of cortical bone density.

The mean acetabular inclination was 41° ± 9° (range 29–59°) and the mean combined anteversion was 39° ± 11° (range 13–54°). At radiographic evaluation, cup radiolucent lines (RLLs) were observed in three cases (3.3%). In all of the cases, cup RLLs were observed in the DeLee and Charnley zone two and were <2 mm. Nonetheless, no cases of cup loosening were observed. A decrease in periacetabular cancellous bone density with retention of cortical bone density compatible with retroacetabular stress shielding was observed in 39 patients (43.0%) (Figure 4). Heterotopic calcifications were present in six patients (6.8%). No cases of femoral notching or liner malseating were observed. Subgroup analysis between PPS and OsseoTi cup coatings showed no significant differences in radiographic findings.

## 4. Discussion

To date, this is the first study investigating this modular dual mobility design survivorship, complications, clinical and radiographic outcomes in primary total hip arthroplasty. The main finding of the study is that the Zimmer G7 modular dual mobility cup appears to be a safe and effective option and does not present specific implant-related mechanical or biological issues in primary total hip arthroplasty at a minimum two-year follow-up.

In this series, modular dual mobility revision-free survival was observed to be 98.0%. Accordingly, Schaffler et al. report modular dual mobility acetabular components survivorship to be 97.6% at a minimum two-year follow-up [21]. Ruusiala et al. observed modular dual mobility revision-free survival to be 97% in primary THA at two- and three-year follow-up [22]. In this series, one patient was revised because of a periprosthetic infection, and one patient was revised for instability. Historically, a higher incidence of cup loosening was observed for dual mobility constructs compared to standard cups; this higher mobilization rate was suggested to be related to the non-bioactive surface coatings, which did not allow osseointegration, but caused the formation of fibrotic tissue instead of new bone [23,24,25]. In this series, no case of aseptic cup loosening was reported. This could be due to the short-term follow-up and to the new-generation materials used. In particular, a highly porous titanium coating (OsseoTi Porous Structure) was used whenever the acetabular bone quality was suboptimal in order to facilitate bone ingrowth and improve biological fixation and survival over time [26]. Furthermore, the modular dual mobility allowed for supplementary screw fixation if press-fit stability was in question. Lastly, a vitamin E-stabilized highly crosslinked polyethylene mobile liner was used in all of the cases to increase wear resistance and reduce osteolytic particle debris [27].

Traditionally, mechanical issues such as liner malseating, femoral notching and intraprosthetic dislocation have been described for dual mobility acetabular cups. In particular, Hamadouche et al. reported a 2.4% incidence of IPD in a series of 168 monoblock DM cups [28], and Schwartz et al. described three cases of IPD in a prospective study of 121 monoblock DM cups [29]. In other studies, the incidence of intraprosthetic dislocation was observed to range from 0 to 5.2% [30,31,32,33]. Traditionally, intraprosthetic dislocation has been considered to be secondary to the degeneration of the retention margin of the polyethylene insert [34]. In order to reduce this wear, the retentive edge gas is chamfered and the polyethylene is enriched with vitamin E in order to reduce the processes of degradation due to oxidation [35,36]. In this series, no intraprosthetic dislocation was observed. Among other MDM mechanical complications, liner malseating and femoral notching secondary to neck impingement with the dual mobility shell rim have been reported [9,37]. At a mean radiographic follow-up of 1.3 years, Lygrisse et al. observed a 3.5% incidence of femoral notching in cylindro-spherical MDM cups [9]. Indeed, cylindro-spherical MDM designs were associated with a decreased impingement-free range of motion, which was further influenced by abnormal spino-pelvic mobility and suboptimal cup positioning, which can be more frequent in dual mobility constructs because of their increased stability [9]. In this series, no cases of femoral notching were reported. This could be secondary to the design of the modular dual mobility liner, which sits flush to the cup, and to positioning the cup in a compliant anteversion to the stem antetorsion by exploiting the “femur first” technique, which has been demonstrated to avoid components impingement [14]. Moreover, no cases of liner malseating were observed in this series. This could be due to this modular dual mobility design surgical technique, which provides a liner inserter ring to align the taper interface for the proper seating of the CoCr liner into the shell and to avoid liner malseating.

Biological issues have been reported for modular dual mobility: fretting corrosion between the cobalt-chrome liner and the titanium cup was described [6,7,8]. These issues could lead to CoCr debris generation with the associated risk of elevated blood metal ions levels and adverse local tissue reactions (ALTR) [9,10,11]. In particular, Abdelaal et al. observed severe corrosion on the backside of the modular acetabular liner and the inner surface of the titanium shell during a revision total hip arthroplasty for intraprosthetic dislocation in a patient with asymptomatic elevated serum cobalt levels [38]. Similarly, Sonn et al. reported a series of three patients with mechanically assisted crevice corrosion at the acetabular component-metal dual mobility liner interface [39]. Moreover, ceramic femoral heads were used in 92% of cases in this series. The predominance of ceramic heads may have reduced the potential for taper fretting and corrosion, compared with cobalt-chrome heads, thereby potentially mitigating the biological risks associated with modular dual mobility constructs [40,41]. In the present study, no patient was revised for adverse local tissue reactions. In the two revised patients, no component corrosion and no metallic debris and/or adverse local tissue reactions were observed. However, blood metal ions levels and MRI imaging to evaluate soft tissue status were not routinely obtained at follow-up.

Dual mobility allows for a lower total hip arthroplasty dislocation risk. According to a deep learning calculator used to predict hip arthroplasty dislocation risk, DM acetabular constructs markedly decrease dislocation risk in primary THA, independently from implant position, spine surgery and spinopelvic alignment [42]. Jones et al. reported only one case of traumatic dislocation and consequent intraprosthetic dislocation, following a closed reduction attempt on a series of 151 minimally invasive posterolateral primary THAs at a high risk of dislocation [5]. In this series, only one posterior dislocation was observed. The increased stability of dual mobility constructs is secondary to a better head–neck ratio, an increase in jump distance and in the range of motion prior to impingement [5]. This is not true for every modular dual mobility design. In particular, whenever the center of rotation was lateralized with respect to the cup opening plane (positive head offset), modular dual mobility was observed to increase the range of motion, but to reduce jump distance and hip stability parameters compared to monoblock dual mobility [43]. Accordingly, Sariali et al. observed that head offset was the most important parameter influencing the jumping distance in total hip arthroplasty [44]. This design instead does not lateralize the head center of rotation, which lies on the cup opening plane (neutral head offset).

Modular dual mobility primary total hip arthroplasty is an effective procedure, since significant improvements were observed in clinical scores in this cohort, independently of the surgical indication. Historically, dual mobility clinical outcomes were affected by a greater incidence of iliopsoas tendinitis [8,9,45,46]. This was reported to be secondary to anterior soft-tissue impingement with the large diameter mobile liner and to the greater stability of the construct potentially leading to insufficient cup anteversion [8,9,43,44]. Moreover, dual mobility shells can be cylindro-spherical in shape and might protrude from the acetabular socket, resulting in anterior soft-tissues impingement [8,9,43,44]. Only one case of iliopsoas tendinitis was reported in the included series. This could be due to the smaller mobile liner diameters required to fit into the modular dual mobility CoCr liner and the standard acetabular cup. Then, iliopsoas impingement was observed to be associated with younger age [47]; as the mean age in the clinically reviewed patients was 73.6 years, this patient cohort had lower functional demands. Moreover, a postero-lateral surgical approach was selected for all the included patients. Anterior or anterolateral surgical approaches were observed to carry a higher incidence of iliopsoas impingement compared to posterior approaches, because of the reduced cup anteversion meant to increase stability and the anterior capsule excision, which could increase the exposed acetabular metal area [48].

A decrease in periacetabular cancellous bone density with retention of cortical bone density compatible with retroacetabular stress shielding was observed in 43.0% of the patients. Retroacetabular stress shielding was defined as periacetabular cancellous bone remodeling with retention of cortical bone density because of stress transfer to the cortical bone [49,50]. Indeed, using finite element analysis, Huiskes et al. investigated the load transfer mechanism and stress patterns of an uncemented threaded cup in the acetabular bone and reported that the shell behaves as a rigid implant by shielding the cancellous bone and enhancing load transfer to the cortical bone [51]. Accordingly, a quantitative computed tomography osteodensitometry analysis observed that the density of cancellous bone decreased by 35% in the retroacetabular pubis, 30% in the ischium, and 18% in the ilium region, while ilium cortical bone density increased by 4% [52]. Similarly, using dual-energy X-ray absorptiometry, Digas et al. observed that bone density was reduced proximally and medially in uncemented porous-coated press-fit cups with hard bearings at a two-year follow-up [53]. Since acetabular components designed for insertion with hard liners need a solid metal shell with high structural stiffness, it is unclear whether a hard CoCr liner would further increase the stiffness of the construct and influence stress transfer to the acetabulum and pelvic bone and subsequent periacetabular bone remodeling.

This study presents the following strengths: the series included all consecutive unilateral total hip arthroplasty prospectively enrolled in the same center during the index period; the same implant and the same technique were used in all cases; and radiographic and clinical evaluation was performed by two orthopedic specialists trained in hip surgery. However, this study also presents some limitations. First, its retrospective nature lowers its level of evidence and does not permit an analysis of the progressive evolution of clinical and radiographic outcomes. Secondly, it lacks a control group; to confirm our findings, a comparative radiographic study including modular dual mobility and standard bearing surface would be needed. Third, indications for surgery were heterogeneous (including osteoarthritis, avascular necrosis, post-traumatic arthritis, acute femoral neck fracture, and dysplasia), and femoral reconstruction was performed using different fixation methods (cemented and uncemented) and stem designs. These factors may act as potential confounders, limiting the ability to attribute outcomes exclusively to the modular dual mobility acetabular construct. Plus, some patients were lost to follow-up due to death, impossibility to contact or inability to participate due to health status, leaving a limited number of participants, compared to the original cohort. However, considering the advanced age of the population analyzed, patient loss is an inevitable consequence, similarly to other series. Then, using crude event proportion as measure of the event burden in reporting rates could lead to an underestimation of the event burden, in case events have not occurred yet by the time of the most recent follow-up examination. Moreover, it remains difficult to determine whether the absence of femoral notching and intraprosthetic dislocation is attributable to implant geometry, the femur-first technique, or patient spinopelvic alignment, which was not systematically assessed. Lastly, fretting corrosion with the associated risk of elevated blood metal ion levels and adverse local tissue reactions was described as a potential biological issue of modular dual mobility acetabular cups, but blood metal ion levels and MRI imaging to evaluate soft tissue status were not routinely obtained at follow-up. The absence of ALTR-related revisions in our cohort cannot substitute for active surveillance, particularly given the relatively short mean follow-up of 2–3 years. Indeed, the mean follow-up of 2.5 years is relatively short and may be insufficient to detect corrosion-mediated soft-tissue reactions or mid-term implant loosening, which often manifest later. Longer-term studies are therefore needed to better assess the biological safety and durability of modular dual mobility constructs.

## 5. Conclusions

This study suggests that the Zimmer G7 modular dual mobility acetabular system provides reliable short-term survivorship, with low complication and revision rates, and significant clinical improvement in patients undergoing primary total hip arthroplasty. No implant-related mechanical failures, intraprosthetic dislocations, or aseptic loosening were observed, and radiographic outcomes were satisfactory at a mean follow-up of 2.5 years. However, concerns regarding potential long-term biological complications, particularly corrosion-mediated adverse local tissue reactions, cannot be excluded based on the current follow-up and lack of systematic surveillance with metal ion testing or soft-tissue imaging. Larger comparative studies with longer follow-up are required to confirm the longevity and biological safety of modular dual mobility constructs in primary total hip arthroplasty.

## Figures and Tables

**Table 1:** Implanted acetabular components sizes and implanted head sizes and related materials.

Cup Size (mm)	Cases	Percentage (%)
46	8	7.6
48	27	25.7
50	31	29.5
52	16	15.2
54	10	9.5
56	6	5.7
58	1	1.0
60	4	3.8
62	2	1.9
**Total**	105	100.0
		
**Liner size (mm)**	**Cases**	**Percentage (%)**
36	8	7.6
38	27	25.7
40	31	29.5
42	16	15.2
44	16	15.2
46	5	4.8
50	2	1.9
**Total**	105	100.0
		
**Head size (mm)**	**Cases**	**Percentage (%)**
22	8	7.6
28	97	92.3
**Total**	105	100.0
		
**Head material**	**Cases**	**Percentage (%)**
CoCr	8	7.6
Ceramic	97	92.3
**Total**	105	100.0

**Table 2:** Femoral stems designs and related femoral stem implants.

Stem	Implant	Cases	Percentage (%)
Standard cementless stem		69	65.7
	CLS Spotorno Hip Stem 135	45	42.9
	CLS Spotorno Hip Stem 125	24	22.9
Conical tapered cementless stem		5	4.8
	Wagner Cone 125	2	1.9
	Wagner Cone 135	3	2.8
Cemented stem		31	29.5
	MS30 standard neck	31	29.5
Total		105	100.0

**Table 3:** Baseline characteristics of patients reviewed at ≥2 years (n = 89) and those who were lost to follow-up, revised or deceased (n = 16).

Group	Reviewed (n = 89)	Lost or Deceased (n = 16)
**N. of patients**	89 (100%)	16 (100%)
**Mean age**	73.5	76.4
**Gender**	62 F (69.7%)	11 F (68.7%)
	27 M (30.3%)	5 M (31.3%)
**Head material**	82 CER (92.1%)	15 CER (93.7%)
	7 MET (7.9%)	1 MET (6.3%)
**Cemented/Cementless stem**	25 Cemented (30.3%)	6 Cemented (37.5%)
	64 Cementless (69.7%)	10 Cementless (62.5%)
**OsseoTi/PPS**	30 OsseoTi (33.7%)	8 OsseoTi (50.0%)
	59 PPS (66.3%)	8 PPS (50.0%)
**Primary osteoarthritis**	69 (77.5%)	14 (87.5%)
**Avascular necrosis**	10 (11.2%)	1 (6.3%)
**Post-traumatic osteoarthritis**	5 (5.6%)	0
**Femur neck fracture**	1 (1.1%)	1 (6.3%)
**Neck fracture sequelae**	1 (1.1%)	0
**Girdlestone sequelae**	1 (1.1%)	0
**Adult hip dysplasia**	2 (2.2%)	0

## Data Availability

The original contributions presented in this study are included in the article. Further inquiries can be directed to the corresponding author(s).
